# Perspectives on AI and Novel Technologies Among Older Adults, Clinicians, Payers, Investors, and Developers

**DOI:** 10.1001/jamanetworkopen.2025.3316

**Published:** 2025-04-04

**Authors:** Nancy L. Schoenborn, Kacey Chae, Jacqueline Massare, Sato Ashida, Peter Abadir, Alicia I. Arbaje, Mathias Unberath, Phillip Phan, Thomas K. M. Cudjoe

**Affiliations:** 1 Division of Geriatric Medicine and Gerontology, Department of Medicine, Johns Hopkins University School of Medicine, Baltimore, Maryland; 2 Center on Aging and Health, Johns Hopkins University, Baltimore, Maryland; 3 Division of General Internal Medicine, Department of Medicine, Johns Hopkins University School of Medicine, Baltimore, Maryland; 4 Department of Community and Behavioral Health, University of Iowa College of Public Health, Iowa City; 5 Department of Computer Science, Johns Hopkins Whiting School of Engineering, Baltimore, Maryland; 6 Johns Hopkins Carey Business School, Baltimore, Maryland

## Abstract

**Importance:**

Artificial intelligence (AI) and novel technologies, such as remote sensors, robotics, and decision support algorithms, offer the potential for improving the health and well-being of older adults, but the priorities of key partners across the technology innovation continuum are not well understood.

**Objective:**

To examine the priorities and suggested applications for AI and novel technologies for older adults among key partners.

**Design, Setting, and Participants:**

This qualitative study comprised individual interviews using grounded theory conducted from May 24, 2023, to January 24, 2024. Recruitment occurred via referrals through the Johns Hopkins Artificial Intelligence and Technology Collaboratory for Aging Research. Participants included adults aged 60 years or older or their caregivers, clinicians, leaders in health systems or insurance plans (ie, payers), investors, and technology developers.

**Main Outcomes and Measures:**

To assess priority areas, older adults, caregivers, clinicians, and payers were asked about the most important challenges faced by older adults and their caregivers, and investors and technology developers were asked about the most important opportunities associated with older adults and technology. All participants were asked for suggestions regarding AI and technology applications. Payers, investors, and technology developers were asked about end user engagement, and all groups except technology developers were asked about suggestions for technology development. Interviews were analyzed using qualitative thematic analysis. Distinct priority areas were identified, and the frequency and type of priority areas were compared by participant groups to assess the extent of overlap in priorities across groups.

**Results:**

Participants included 15 older adults or caregivers (mean age, 71.3 years [range, 65-93 years]; 4 men [26.7%]), 15 clinicians (mean age, 50.3 years [range, 33-69 years]; 8 men [53.3%]), 8 payers (mean age, 51.6 years [range, 36-65 years]; 5 men [62.5%]), 5 investors (mean age, 42.4 years [range, 31-56 years]; 5 men [100%]), and 6 technology developers (mean age, 42.0 years [range, 27-62 years]; 6 men [100%]). There were different priorities across key partners, with the most overlap between older adults or caregivers and clinicians and the least overlap between older adults or caregivers and investors and technology developers. Participants suggested novel applications, such as using reminders for motivating self-care or social engagement. There were few to no suggestions that addressed activities of daily living, which was the most frequently reported priority for older adults or caregivers. Although all participants agreed on the importance of engaging end users, engagement challenges included regulatory barriers and stronger influence of payers relative to other end users.

**Conclusions and Relevance:**

This qualitative interview study found important differences in priorities for AI and novel technologies for older adults across key partners. Public health, regulatory, and advocacy strategies are needed to raise awareness about these priorities, foster engagement, and align incentives to effectively use AI to improve the health of older adults.

## Key Points

**Question:**

What are the priority areas and suggested uses for artificial intelligence (AI) and novel technologies for older adults among key partners including older adults, caregivers, clinicians, payers, investors, and technology developers?

**Findings:**

A qualitative interview study comprising 49 individuals found that the priority areas ranged from daily care to systemic infrastructure, with a misalignment in priorities across different types of key partners and with challenges for optimal engagement. Suggested applications included novel extensions of existing technology, which can inform future product development.

**Meaning:**

This study suggests that strategies are needed to better engage key partners across the continuum of AI technology development and align priorities.

## Teaser

This qualitative interview study examines the priorities and suggested applications for artificial intelligence (AI) and novel technologies for older adults among key partners including older adults, caregivers, clinicians, payers, investors, and technology developers.

## Introduction

The population of older adults is growing, and many older adults’ care needs are not adequately or equitably met.^1,2,3,4^ Emerging technologies have the potential to improve the health and care of older adults.^5,6^ A range of artificial intelligence (AI) products coupled with novel technologies, such as remote sensors, robotics, and decision support algorithms, have been proposed or developed for older adults,^7,8,9,10,11,12,13,14^ such as the use of machine learning for predicting fall risk and robotics or avatars to enhance social engagement.^8,9,10^

Multiple key partners are relevant for the development and implementation of novel technologies.^15^ They include developers who conceive the innovation, investors who finance the product, health system leaders and payers who adopt the product, and older adults, caregivers, and clinicians who are the end users. Little is currently known about these key partners’ perspectives regarding the use of AI and novel technologies for older adults. Better understanding these perspectives can help ensure that future innovations meet the needs of key partners.^16,17^ Understanding the extent of agreement of priorities across key partner groups can inform strategies to proactively align incentives and facilitate engagement.

Prior studies have often focused on the perceived benefits and concerns associated with AI rather than priorities for future development.^15,18,19,20,21^ Prior studies have also not focused on older adults,^15,18,19,20,21,22^ who likely have unique considerations in terms of priorities for technology in health care and suggested applications. Last, prior studies have focused on members of the public, clinicians, and AI researchers but have not included payers or investors,^15,18,19,20,21,22,23^ who are instrumental in financing, developing, and implementing technologies in health care.

To address the knowledge gap, we examined perspectives from older adults, caregivers, clinicians, health system or health insurance leaders, technology investors, and technology developers. We examined perspectives regarding the use of AI and novel technologies to improve the health and well-being of older adults, focusing on priority areas, suggested applications, and key partner engagement.

## Methods

### Design, Setting, and Recruitment

This project, conducted from May 24, 2023, to January 24, 2024, originated from the Johns Hopkins Artificial Intelligence and Technology Collaboratory for Aging Research (JHU-AITC).^5,24^ We chose the qualitative study design of grounded theory to gather new knowledge about key partner priorities to develop new approaches to AI and novel technology in the future.^25^ Data collected via semistructured individual interviews. All participants needed to be English speaking and provide informed oral consent. Each participant was offered a $50 gift card. This project was approved by the Johns Hopkins School of Medicine institutional review board. Study reporting followed the Consolidated Criteria for Reporting Qualitative Research (COREQ) reporting guideline.

Recruitment was purposively stratified to sample key informants from 5 groups^26^: (1) adults aged 60 years or older and caregivers for someone aged 60 years or older (these groups were combined because many older adults were or had been caregivers); (2) clinicians who cared for adults aged 60 years or older; (3) health system or health insurance leaders who were involved in decisions about technology adoption, such as Chief Informational Officers, Chief Medical Officers, or Chief Quality Officers (hereafter referred to as *payers*); (4) investors in aging-related technology or AI; and (5) technology developers in aging-related technology or AI. As payers, investors, and technology developers may have experiences in more than 1 category, we asked the participants to self-identify the single category that best represented their perspective.

Within each participant category, recruitment occurred through a combination of maximal variation and snowball sampling.^26^ JHU-AITC engages with a stakeholder council of older adults, caregivers, and clinicians from Maryland and Iowa. Recruitment of older adults, caregivers, and clinicians occurred through referrals from the study team and council members. We used maximum variation sampling to target individuals diverse in age, sex, race and ethnicity, and rural and urban locations. Race and ethnicity were self reported and were assessed to provide a more comprehensive description of the study population. The race and ethnicity categories were provided as questionnaire options to the participants, but they could also choose other and elaborate; the specific options were the following: American Indian or Alaska Native, Asian, Black or African American, Hispanic or Latino, Native Hawaiian or Other Pacific Islander, White, or other. For clinicians, we also aimed to diversify practice settings and disciplines. A total of 7 of 16 council members and 23 of 40 referred older adults, caregivers, and clinicians participated. Recruitment of payers, investors, and technology developers focused on key informants with the experiences or roles, as outlined in eligibility criteria, who were identified by the study team, JHU-AITC colleagues, and the coordinating center that supports Artificial Intelligence and Technology Collaboratories (AITCs). A total of 8 of 8 referred payers, 5 of 7 referred investors, and 6 of 9 referred technology developers participated. Recruitment continued until data saturation was reached within each category.^27^

### Interview Guide

The interview guide was developed by the study team, which included individuals with expertise in engineering, business development, patient engagement, and geriatric medicine, in an iterative fashion. The interview guide was informed by a systematic review in which the first stage of stakeholder involvement was identification of research priorities.^16^ We assessed priorities by asking about the most important problems faced by older adults and caregivers, or the most important opportunities associated with older adults and AI or novel technologies, and all groups except technology developers were asked about suggestions for technology development; then, we asked for suggestions of technology solutions. Given the study focus on key partner engagement, we also asked about current practices around engagement. We defined AI as “technologies where machines try to do what human beings do” and described examples of AI applications such as alerts, sensors, prediction algorithms, or robots (see the eAppendix in Supplement 1 for the full interview guide).

### Data Collection and Analysis

Three team members (including N.L.S.) with prior qualitative research experience conducted interviews in person (at private work spaces) or virtually from May 24, 2023, to January 24, 2024. One team member (N.L.S.) had prior working relationships with some of the clinician participants.

Interviews were audio-recorded and transcribed verbatim; durations ranged from 12 to 69 minutes. We continuously and iteratively reviewed the transcripts concurrent with data collection. We determined that data saturation had been reached within each participant category when no new ideas were emerging.^27^

Prior to the interviews, we collected information via a structured questionnaire on age, sex, race and ethnicity, and zip code. For older adults or caregivers, we also asked about educational level, financial strain,^28^ health literacy,^29^ self-rated health, and duration of caregiving. For all other participants, we asked about professional roles and duration working in that role. For clinicians we also asked about the proportion of older adult patients.

Analysis was stratified by key partner type. We used thematic analysis to code the transcripts using the ATLAS.ti, version 24, software (ATLAS.ti Scientific Software Development GmbH).^30,31^ A preliminary codebook was developed that included deductive codes based on the interview guide. Three investigators (N.L.S., K.C., and J.M.) independently reviewed 12 randomly selected transcripts, including 1 to 3 transcripts from each participant category, to inductively generate additional codes. These analyses took the forms of open coding (line-by-line examination of the data to generate concepts), memo writing (to track analysis and minimize introduction of preconceived notions), axial coding (to identify relationships among codes and reorganize codes), and constant comparison (to identify similarities and differences across code groups and across participant groups).^31^ The inductively generated codes were added to the codebook. Then, the 3 investigators coded 3 transcripts together using the revised codebook to ensure that the code definitions were clear and consistently applied. At this stage, the codebook was finalized. Each of the remaining 46 transcripts was coded independently by at least 2 investigators (N.L.S., K.C., or J.M.) and differences were reconciled by consensus.

**Figure 1.**
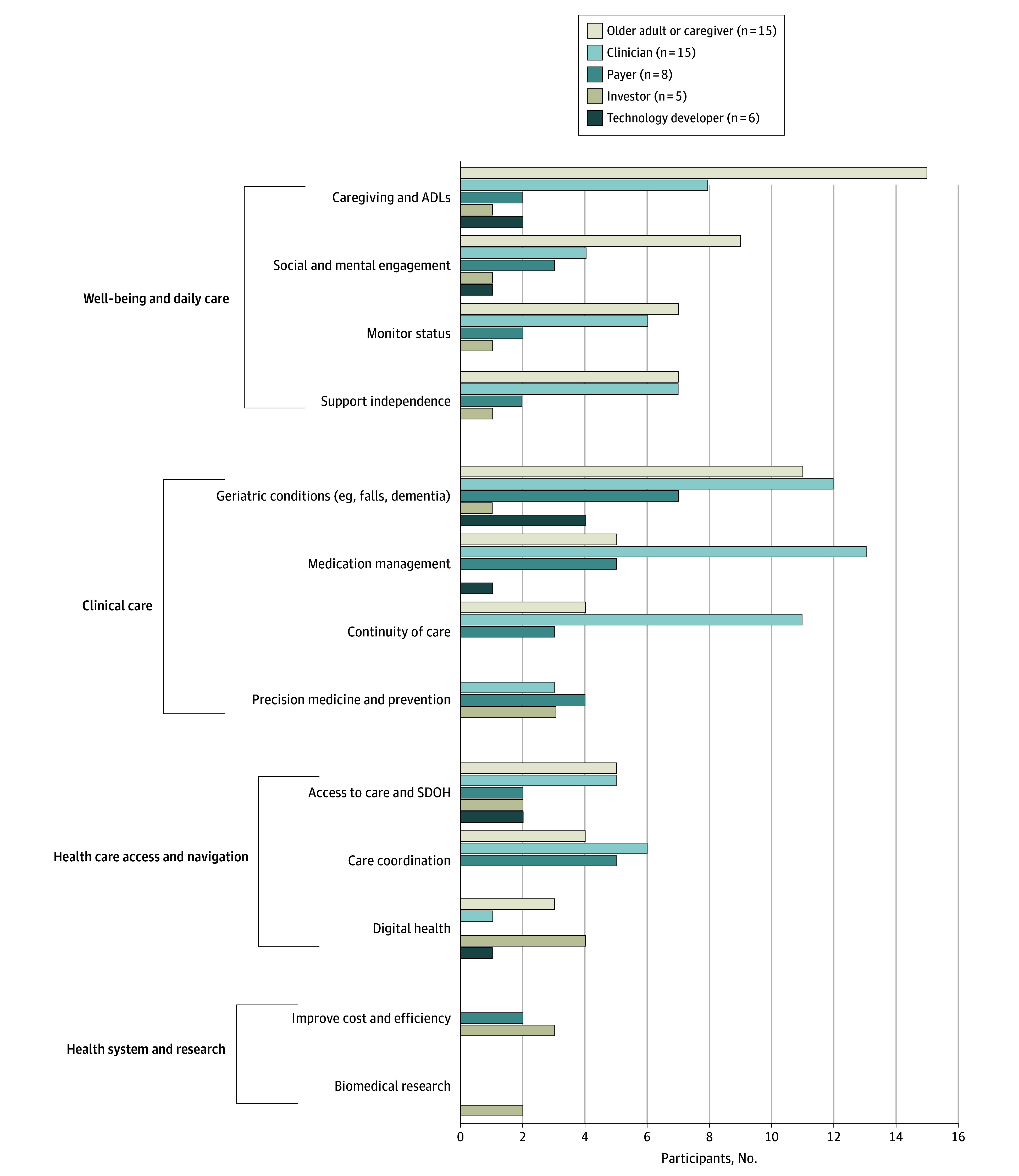
Frequency of Priority Areas Mentioned by Participants Regarding Artificial Intelligence and Novel Technology Use in the Care of Older Adults, Stratified by Key Partner Group ADLs indicates activities of daily living; SDOH, social determinants of health.

**Figure 2.**
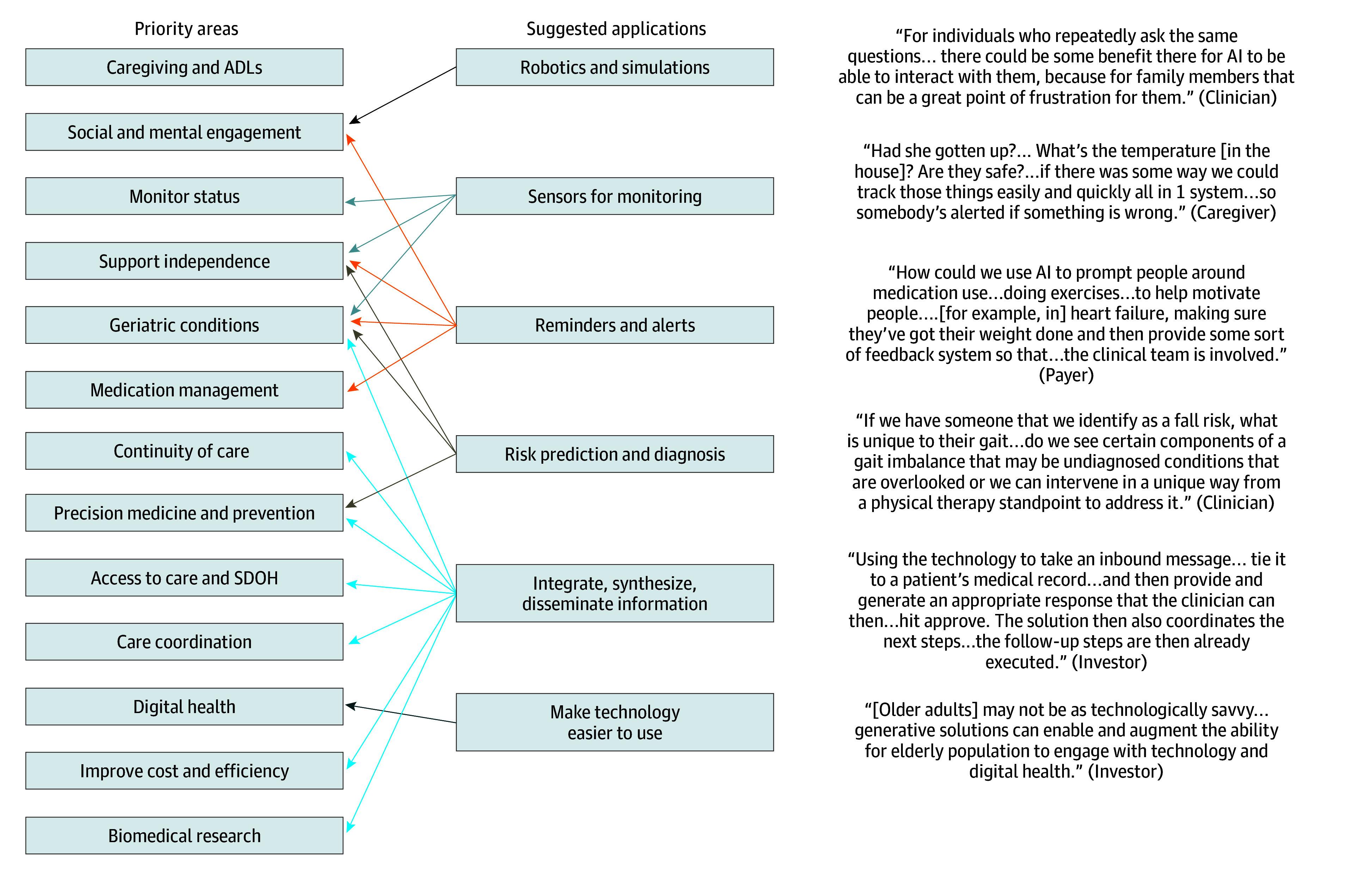
Suggested Artificial Intelligence (AI) and Novel Technology Applications by Priority Areas With Illustrative Quotes Arrows signify participants’ suggestions of using an application to address a specific priority area. Arrows for each suggested application are given a different color for clearer visualization. ADLs indicates activities of daily living; SDOH, social determinants of health.

We summarized results into major themes within each of the 3 study topics: priority areas, suggested applications, and key partner engagement. For priority areas, we compared the frequency and type of priority areas by participant group to assess the extent of overlap across groups (Figure 1). We also assessed the co-occurrence of suggested applications with the priority areas (Figure 2). We used representative quotes for illustration.

## Results

The 49 participants included 15 older adults or caregivers (mean age, 71.3 years [range, 65-93 years]; 4 men [26.7%] and 11 women [73.3%]; 1 Asian participant [6.7%], 3 Black participants [20.0%], and 11 White participants [73.3%]), 15 clinicians (mean age, 50.3 years [range, 33-69 years]; 8 men [53.3%] and 7 women [46.7%]; 3 Asian participants [20.0%], 1 Hispanic participant [6.7%], and 11 White participants [73.3%]), 8 payers (mean age, 51.6 years [range, 36-65 years]; 5 men [62.5%] and 3 women [37.5%]; 8 White participants [100%]), 5 investors (mean age, 42.4 years [range, 31-56 years]; 5 men [100%]; 4 Asian participants [80.0%] and 1 White participant [20.0%]), and 6 technology developers (mean age, 42.0 years [range, 27-62 years]; 6 men [100%]; 1 Asian participant [16.7%], 1 Black participant [16.7%], 3 White participants [50.0%], and 1 mixed race [Middle Eastern and White] participant [16.7%]) (Table 1).^20,21^ A total of 9 individuals in the older adult or caregiver group reported caregiving experience. Clinicians included physicians, nurses, pharmacists, rehabilitation therapists, and a dentist; 14 of 15 clinicians reported caring for mostly older adults. Most of the older adult or caregiver and clinician participants were from Maryland and Iowa, whereas payer, investor, and technology developer participants were from 8 different states (Maryland, Iowa, Colorado, Florida, Massachusetts, Minnesota, California, and Virginia) and Washington, DC.

**Table 1.** Participant Characteristics

Characteristic	Older adult or caregiver (n = 15)	Clinician (n = 15)	Payer (n = 8)	Investor (n = 5)	Technology developer (n = 6)
Age, mean (range), y	71.3 (65-93)	50.3 (33-69)	51.6 (36-65)	42.4 (31-56)	42.0 (27-62)
Sex, No. (%)					
Women	11 (73.3)	7 (46.7)	3 (37.5)	0	0
Men	4 (26.7)	8 (53.3)	5 (62.5)	5 (100)	6 (100)
Race and ethnicity, No. (%)					
Asian	1 (6.7)	3 (20.0)	0	4 (80.0)	1 (16.7)
Black	3 (20.0)	0	0	0	1 (16.7)
Hispanic	0	1 (6.7)	0	0	0
White	11 (73.3)	11 (73.3)	8 (100)	1 (20.0)	3 (60.0)
Mixed race (Middle Eastern and White)	0	0	0	0	1 (16.7)
Location, No. (%)					
Maryland	10 (66.7)	8 (53.3)	2 (25.0)	0	3 (50.0)
Iowa	4 (26.7)	7 (46.7)	2 (25.0)	0	0
Other states (as listed)	1 (6.7): DC	0	4 (50.0): CO, FL, MA, MN	5 (100.0): CA, MA, VA	3 (50.0): DC, VA
Past or current caregiver	9 (60.0)	NA	NA	NA	NA
No. of years as caregiver, mean (range)	9.5 (0.5-25)	NA	NA	NA	NA
Educational level, No. (%)					
College or higher degree	3 (20.0)	NA	NA	NA	NA
High school or <4 y college	11 (73.3)	NA	NA	NA	NA
Missing data	1 (6.7)	NA	NA	NA	NA
Self-reported financial strain, No. (%)^a^	3 (20.0)	NA	NA	NA	NA
Low health literacy, No. (%)^b^	3 (20.0)	NA	NA	NA	NA
Self-rated health, No. (%)					
Excellent or very good	6 (40.0)	NA	NA	NA	NA
Good	4 (26.7)	NA	NA	NA	NA
Fair or poor	4 (26.7)	NA	NA	NA	NA
Missing	1 (6.7)	NA	NA	NA	NA
Clinician role, No. (%)					
Physician	NA	6 (40.0)	NA	NA	NA
Nurse	NA	4 (26.7)	NA	NA	NA
Pharmacist	NA	2 (13.3)	NA	NA	NA
Rehabilitation therapist^c^	NA	2 (13.3)	NA	NA	NA
Dentist	NA	1 (6.7)	NA	NA	NA
Clinician practice setting (can choose >1), No. (%)					
Inpatient	NA	10 (66.7)	NA	NA	NA
Outpatient	NA	5 (33.3)	NA	NA	NA
Nursing home	NA	3 (20.0)	NA	NA	NA
Home-based care	NA	4 (26.7)	NA	NA	NA
≥70% of Patients aged ≥65 y, No. (%)	NA	14 (93.3)	NA	NA	NA
Duration in profession, mean (SD), y	NA	19.3 (11.1)	13.3 (9.5)	4.5 (3.3)	15.2 (13.6)

Abbreviation: NA, not asked for a specific key partner group.

^a^ Financial strain was assessed using the question: “During the last year, were there any times when you did not have enough money to pay rent or utilities or medical bills?” Response “yes” was counted as having self-reported financial strain.^20^

^b^ Low health literacy was assessed using the question: “How confident are you in filling out medical forms by yourself?” Responses of “somewhat,” “a little bit,” and “not at all” were counted as low health literacy.^21^

^c^ Rehabilitation therapist included physical therapist and occupational therapist.

### Topic 1: Priority Areas

Distinct priorities were identified across key partner groups. The reported priorities for AI and novel technology use among older adults were summarized into multiple areas, ranging from daily care to research infrastructure (Table 2; Figure 1). Although there was some overlap in priorities across key partner groups, there were also distinct perspectives within each category.

**Table 2.** Priority Areas Identified by Participants, With Illustrative Quotes, Regarding AI and Novel Technology Use in the Care of Older Adults

Priority area	Illustrative quotes
Caregiving or activities of daily living	I have problems getting around… I have a very bad back, so it’s kind of challenging getting around and doing things. I have to really be careful. (Older adult) We were worried about food poisoning. Things that should have been refrigerated that weren’t… or in the refrigerator way too long… And then hygiene and incontinence, both bladder and bowel… was causing cleaning problems. (Caregiver)
Social or mental engagement	When you lose a spouse… the main thing that I hear from people is the extreme loneliness… So I see AI… could be providing some companionship… to combat loneliness. (Older adult) Watching something on a screen… that’s not what they want. They need something that they love to do and is more personalized… and interactive. (Caregiver)
Monitor status	I had a security camera in the living room that could watch him, so I was watching my phone constantly, even in the bathroom. I had the volume turned up just so I could hear him move… I wish there was some kind of AI that could say “Daddy’s getting up out of the chair now… Daddy is about to fall.” (Caregiver)
Support independence	Keep them independent in their home. They want to live in their home… anything we can do to keep someone living in the home they are familiar with… is hugely important. (Clinician)
Geriatric conditions (ie, dementia, falls)	A lot of people are going after cognitive issues, Alzheimer’s dementia–related innovation and technologies. (Technology developer)
Medication management	Medication usage and when do you use it is definitely a big problem. What they tell me they take, I’m not too sure whether it really is true or not… even if the caregiver puts out the medication, sometimes I don’t think it’s taken. (Clinician)
Continuity of care	One very simple low-hanging fruit for use of technology is to integrate multiple databases… already-existing databases, so the dental, the medical, the nursing home, the social services databases… and connect people with the appropriate providers. (Clinician)
Precision medicine or prevention	Another [opportunity] area… is precision medicine, things that are more targeted for an individual basis… to personalize every individual patient’s approach with different types of treatments… to achieve better outcomes. (Investor)
Access to care or social determinants of health	How do we address chronic conditions… activities of daily living, social determinants of health, so that would be food, transportation, housing. (Payer)
Care coordination	Helping with navigation… support being connected to the services that I need that are paid for by the insurance… linking to community support services, that linkage also identifies those services that have capacity, that are high quality. (Payer)
Digital health	More about digital health, using digital tools in health care to get them cared for in the setting that they choose, to use digital tools to have more self-care. (Investor)
Improve cost or efficiency	The cost of health care is super expensive and we are always mindful… there’s a balance of providing as much service as possible but keeping the cost as low as possible. (Payer)
Biomedical research	We want to provide the tools for biology development, whether it is medical devices… or therapeutics development would be very interesting to us. (Investor)

Abbreviation: AI, artificial intelligence.

#### Older Adults, Caregivers, and Clinicians

The highest degree of overlap in priorities was among older adults or caregivers and clinicians. Both groups prioritized activities of daily living (ADLs) or caregiving, geriatric conditions such as falls and dementia, monitoring patient status, and supporting independence. Older adults or caregivers more often mentioned nonclinical aspects of care; in particular, supporting ADLs was an important priority for all older adult and caregiver participants. Clinicians often focused on clinical aspects of care, such as continuity of care and medication management. Older adults with caregiving experience spoke about their own needs as well as caregiving needs. For example, some mentioned monitoring their own health as a priority, whereas others described monitoring the status of a family member as a caregiver.

#### Payers

Payers more frequently identified priorities associated with population health, including care coordination and management of common geriataric conditions. One payer talked about navigating health care resources: “People don’t know where to get information and when they do, they often are flooded with lots of information that has very little meaning… whether it’s selecting a health plan… or looking for community resources… you don’t know how to match what your needs are.”

#### Investors

Investors had little overlap in their priorities with other groups and commented on using AI to improve digital health, increase health care efficiency, and enhance biomedical research. One investor commented about drug development: “The cycle is hugely ineffective, and it’s very expensive; …we want to look at if there’s anything that can shorten the timeline, make a delivery more efficient, and improve the clinical trials results.”

#### Technology Developers

Technology developers reported that the driving forces behind their projects were the revenue stream, pursuit of innovation, and personal experience. One engineer said: “I am working on this project because that’s what I’m being funded to work on. If there’s more funding… for aging, everybody in the lab would work on it.” An engineer and entrepreneur commented: “You get into the question—is there enough return on investment to make that a business or not?” When asked what may attract vs discourage technology developers from focusing on older adults, 1 engineer said: “a lot of times people think geriatrics and they don’t necessarily associate it with innovation.” However, the same participant commented that he became interested in aging and technology because of personal experience: “My grandmother… worked in a nursing home, so I grew up following her around… there was a lot of personal interest.”

### Topic 2: Suggestions

Suggested applications included novel extensions of existing technologies but did not address all priority areas. Participants’ suggested technology applications were summarized into 6 major areas: robotics or simulations, sensors for monitoring, reminders or alerts, risk prediction, information management, and facilitating technology use. These areas were mentioned to address the identified priority areas except for ADLs or caregiving, which was the top priority for older adults or caregivers (Figure 2). Although many of the suggested solutions, such as sensors for monitoring or reminders or alerts for medication adherence, can be helpful for caregivers, very few comments talked about using technology for direct hands-on care or ADLs. These comments came solely from older adult or caregiver participants; they wished for technology that could help in this area but did not specify what that technology may look like. One caregiver said, regarding mobility: “[My father] was in a wheelchair for the last 7 months of his life… I would love it if AI could… help him walk.” A clinician shared the belief that there may not be technological solutions for this area: “Another gizmo is just never [going to] replace the core problem of needing more hands-on care. I don’t see how that could be done by AI.”

Many suggestions corresponded with existing or emerging technologies, but participants also mentioned novel ways to build on the existing technology, such as using reminders and alerts for motivation or for social engagement, using individualized predictions for tailored communication or education and using information synthesis or dissemination to increase health care access.

#### Reminders or Alerts

Participants suggested that AI and novel technologies can do more to provide motivation and coaching. One clinician suggested using AI to tailor a medication reminder that would also provide rationale for the medication to promote adherence: “[We need] in-the-moment communication to the patient why they are taking [the medication], why it’s important.” Another clinician talked about using AI to coach exercise: “My patients who have physical disability… what [they] really want is a coach, someone who’s going to be there and stand over [them] and say: ‘let’s do those leg raises’ and make sure [they] are doing them right and I think AI could do that.”

Participants also commented on using AI to keep track of and remind people about social interactions and engagement goals. One older adult said: “[AI] can talk through what you want to accomplish today, and then [plan] the day and [monitor] whether or not you actually did what you said you would like to do, and if not, then carrying that to next day and remind me… to keep people more engaged in their own well-being and in being able to care for others, like ‘Oh, you didn’t call your husband today. Maybe you should check in with him.’”

#### Individualized Care

Participants commented that, in addition to using AI to personalize disease risk, disease trajectories, or treatment response, AI can also personalize communication and education. One payer said: “Even just thinking about AI algorithms for how people best want to be communicated with and at what intervals. That’d be hugely helpful.” A clinician suggested: “We can use AI… based on your risk factors… to produce personalized educational materials.”

#### Health Care Access

Participants commented that, in addition to using AI to integrate and synthesize information, disseminating the information through clinician support can improve access. One clinician said: “We know that rural communities have a lot less resources… if we have this AI helping providers make decisions or even help as a consultant… this can help a lot of providers in the rural area and expand their capabilities.”

### Topic 3: Engagement

Challenges exist in engaging key partners. Although participants from all key partner groups agreed on the importance of engaging end users, many acknowledged that engagement had been suboptimal. One payer said: “Digital healthcare technology companies have not done a good job of bringing the lens of multiple stakeholders… in their early stages of developing their solution, and also not enough direct feedback from the clinical staff along the way.”

One developer commented that sometimes regulations make partner engagement difficult: “If you want to develop a medical device, you are going to have to go through a lot of red tape to get that to market, to actually really test it out in real life… that means if there’s something that you missed or there’s some inefficiency, you won’t really know for years.”

Participants also commented that, even when key partners were involved, not all voices were weighted equally. One investor said: “There’s certain weights given to different folks, depending on the business model.” There may also be misalignments in incentives between funding sources and the end users. Another investor said: “We want our patients to be happier, [have] better experience, but if that doesn’t turn into immediate dollars, hospitals sometimes are very reluctant to actually pay for these services outside of research.”

As a solution to address this misalignment, 1 payer suggested making key partner priorities more transparent: “A health plan or a health system vs an individual patient, they may have different needs, drivers, priorities… I think you would want to have information from all of them… to help inform the optimal utility of the [product].” Another suggestion was public-private partnership in funding: “Investors can only take it so far… technologies that are making public health impacts… that’s where the government steps in and creates these economics to incentivize development.”

## Discussion

This is the first study, to our knowledge, to examine diverse key partners’ perspectives in the US about their priorities and suggested applications for AI and novel technologies associated with older adults. This study adds to existing literature in several ways. First, prior studies that examined the use of AI in health care and key partner perspectives often focused on general approaches, concerns, or challenges rather than priority setting.^15,18,19,20,21^ The identified priority areas from the current study can critically inform priority setting for technology development among US public health organizations and funding agencies. Second, the inclusion of investors and payers is unique and important because they play key roles in the development and adoption of technologies. Third, prior studies did not often focus on older adults. One study in the UK engaged older adults, clinicians, and AI researchers for AI priority setting but the results may not be generalizable to the US.^23^ The present study identified many suggested uses of AI and other technologies specific to older adults, which is valuable information for entrepreneurs and technology developers.

Participants identified a wide range of priorities with important differences across groups. There was little priority overlap between people responsible for making novel technology available (ie, the developers and investors) and the end users of technology (ie, older adults, caregivers, and clinicians). Recognizing these differences is important for avoiding misdirected efforts and resources that may fail to address the most pressing needs of the end users. One potential explanation for the priority differences is lack of awareness and engagement. Technology developers commented on not being familiar with the needs of older adults and reported that end user engagement had not been optimal in the past. Although it was promising that all participants agreed on the importance of end user engagement, end user engagement when a developer or investor is already working on an innovation can optimize that product but does not address the more fundamental problem of attracting developers and investors to an area in need of innovation. Direct care for ADLs is a high-priority area for older adults and caregivers that has few to no suggested technological solutions and should be the focus of future innovation efforts.

We also found that input from different key partner groups may be weighted differently by technology developers and investors, with stronger influences from the payers. In other words, even if technology developers and investors were made aware of the priorities of end users, they may hesitate to pursue an innovation unless the payer shares the same priorities. This finding highlights the importance of aligning health system and insurance payment incentives with end user needs.

There are several potential solutions to address the priority misalignment among key partner groups. Government and public health agencies can serve an important role in setting priorities and providing incentives to attract innovation. For example, the National Institutes of Health and other government funders can explicitly invite technology innovations focused on priority areas such as ADL support and direct care that have been shown to be important for end users. Regulatory bodies can encourage early-stage end user engagement that is distinct from prototype testing to ensure that end users are involved in the design and development phase of the innovation. For example, the Patient-Centered Outcomes Research Institute (PCORI) requires key partner engagement as an obligatory component of research proposals.^32^ Other funders and regulatory bodies can similarly encourage or require early engagement of end users as part of the criteria for funding or approval of medical devices. Dissemination of best practices for key partner engagement can further facilitate this effort.^33^ Resources are available through PCORI, including an engagement rubric that provides guidance on methods for engaging partners and an engagement plan template with step-by-step instructions.^32^ Organizations that represent the interests of older adults, caregivers, and clinicians can play a role in promoting awareness of their priorities among technology developers and investors. Last, building forums such as AITCs that foster collaboration and engagement across disciplines is essential.

### Strengths and Limitations

Our study has some strengths, including the diverse types of key partners from both urban and rural settings. Out study also has some limitations, including that the older adults or caregivers and clinician participants were mostly from Maryland and Iowa, and their views may not be applicable to other geographic areas. Some key partner groups lacked sex and racial and ethnic diversity. In addition, we included only English speakers. To examine the generalizability of the ideas identified in the current study, we plan a larger, nationally representative survey. Our study design also relied on self-report and may be subject to recall bias and social desirability bias.

## Conclusions

In this qualitative interview study, among diverse key partners, we identified a spectrum of priorities and suggested applications regarding the use of AI and novel technologies for older adults. We found misalignments in the priorities that raise questions on whether societal resources are appropriately directed to address the priorities of end users. Public health, regulatory, and advocacy strategies are needed to raise awareness about these priorities, foster interdisciplinary engagement, and align incentives to ensure that we leverage the full potential of AI and novel technologies to improve care for older adults.

## Notes

Supplement 1. **eAppendix.** Interview Guide

Supplement 2.
Data Sharing Statement
