# Supplementary material for: Perspectives on AI and Novel Technologies Among Older Adults, Clinicians, Payers, Investors, and Developers
Source: JAMA Netw Open. 2025 Apr 4;8(4):e253316. doi: 10.1001/jamanetworkopen.2025.3316 (PMC11971670; doi:10.1001/jamanetworkopen.2025.3316)
Supplement: Supplement 1. — eAppendix. Interview Guide [file jamanetwopen-e253316-s001.pdf]

Supplementary Online Content

Schoenborn NL, Chae K, Massare J, et al. Perspectives on AI and novel technologies among older adults, clinicians, payers, investors, and developers. *JAMA Netw Open.* 2025;8(4):e253316. doi:10.1001/jamanetworkopen.2025.3316

eAppendix. Interview Guide

This supplementary material has been provided by the authors to give readers additional information about their work.

© 2025 Schoenborn NL et al. *JAMA Network Open.*

eAppendix. Interview Guide

Thank you for being part of this study. We would like to understand how to use new technologies to improve the health and well-being of older adults and their caregivers. We are interested specifically in artificial intelligence; Artificial intelligence describes technologies where machines try to do what human beings do. There are many types of AI systems – for example, there are alert or reminder systems to remind people to take medicines, smart watches or wearable sensors that track people’s steps and heart rates, computer algorithms that predict falls based on the data, or robots that provide companionship.

We want to know your opinions about what should be the focus of artificial intelligence, i.e. what problems should artificial intelligence be trying to solve? There is no right or wrong answer.

<u>Older adults/caregivers</u>

(if applicable) We are interested in your perspectives about your own experiences and experiences as a caregiver, so feel free to comment on either or both.

1. What are the most important problems or challenges that you experience related to your health or daily function or when you care for your [relationship to patient]? (PROBE: this could be related to physical, mental, or emotional health) How do you currently deal with these problems?
2. [If applicable: The problems you mentioned are the ones you are experiencing now at home,] we would also like to know what problems you may have experienced when you or your [relationship to patient] was in the hospital, in the emergency room, or skilled nursing facility. Can you tell us about any related experiences? How did you deal with those?
3. Specifically about artificial intelligence. What have you heard about AI? What thoughts do you have about AI? What concerns, if any, do you have about AI?
4. We are trying to understand what health-related problems AI should focus on. We do not expect you to come up with the technology solutions, but if you have any thoughts or suggestions, we’d love to hear them – i.e. are there technologies that you think should be developed to address the problems you mentioned before?
5. What do you want technology developers and AI researchers to know as they work in this area?

© 2025 Schoenborn NL et al. *JAMA Network Open.*

Clinicians

1. Can you please start by sharing a bit about your role in the care of older adults? Such as what setting you practice in and what you do.
2. What are the most important problems or challenges that you experience when caring for older adults? These could be challenges you experience when you care for them or could be what you perceive that the older adults or caregivers are experiencing? (PROBE: this could be related to physical, mental, or emotional health) How do you or they currently handle these problems?
3. [If applicable: The problems you mentioned are the ones experienced in the outpatient or home setting,] we would also like to know what problems you experience in other settings (if you practice there) or that you perceive that older adults or caregivers experience when they are in other settings? How do you or they handle those challenges?
4. Specifically about artificial intelligence. What have you heard about AI? What thoughts do you have about AI? What concerns, if any, do you have about AI?
5. We are trying to understand what problems should AI focus on. We do not expect you to come up with the technology solutions, but if you have any thoughts or suggestions, we'd love to have them – i.e. are there technologies that you think should be developed to address the problems you mentioned before?
6. What do you want technology developers and researchers to know as they work in this area?

© 2025 Schoenborn NL et al. *JAMA Network Open.*

Payers

1. Can you share a little bit of your background in your profession, what is your title and what you do?

2. What are the most important problems or challenges that older adults and caregivers experience (in your health system/health plan/facility/organization)? How are these currently managed?

3. [If applicable: The problems you mentioned are the ones experienced in the outpatient or home setting,] we would also like to know what problems older adults or caregivers may be experiencing in the hospital or in the emergency room?

4. Specifically about artificial intelligence. What have you heard about AI? What thoughts do you have about AI? What concerns, if any, do you have about AI?

5. We are trying to understand what problems should AI focus on. We do not expect you to come up with the technology solutions, but if you have any thoughts or suggestions, we'd love to have them – i.e. are there technologies that you think should be developed to address the problems you mentioned before?

6. What are the top factors you consider when deciding whether or not to adopt/invest in a new technology for the care of older adults? What information or data do you need to make that decision? At what point do you get input from the users?

7. What do you want technology developers and researchers to know as they work in this area?

© 2025 Schoenborn NL et al. *JAMA Network Open.*

Investors

1. Can you share a little bit of your background in your profession, what is your title and what you do?

1. Do you invest in AgeTech? Where do you see the most important or exciting opportunities as investors? What problems in older adults' health and wellbeing can AI or new technologies address the best in your opinion? What are some potential applications that you would love to see or are excited about?

2. What thoughts do you have about AI? What are the benefits? What concerns, if any, do you have about AI? What added value or return on investment do you expect AI to add?

3. What are the key opinion leaders you seek when making investments in Age Tech? Do you include older adults or clinicians? Whose voices matter most?

4. What do you want technology developers and researchers to know about investors' priorities as they work in the AgeTech domain?

5. If older adults and caregivers or clinicians identify the most important problem they want technology to help solve, how might we motivate investors to also focus on those areas?

© 2025 Schoenborn NL et al. *JAMA Network Open.*

Technology developers

1. Can you share a little bit of your background in your profession, what is your title and what you do?

2. Thinking about AI or new technology development, can you tell me how you select an area to focus on? What are some of the most important or exciting areas or ideas in the field? What are some potential applications that you would love to see or are excited about? What would attract people to focus on older adults? What might turn people off from working on products for older adults?

3. What are your thoughts about AI? What are the benefits? What concerns, if any, do you have about AI?

4. At what point during the development process do you get input or feedback from the intended end users? What kind of questions do you ask to receive feedback?

5. If older adults and caregivers or clinicians identify the most important problem they want technology to help solve, how might we motivate technology developers and researchers to then work on these areas?

© 2025 Schoenborn NL et al. *JAMA Network Open.*
