# Supplementary material for: Perspectives on AI and Novel Technologies Among Older Adults, Clinicians, Payers, Investors, and Developers
Source: JAMA Netw Open. 2025 Apr 4;8(4):e253316. doi: 10.1001/jamanetworkopen.2025.3316 (PMC11971670; doi:10.1001/jamanetworkopen.2025.3316)
Supplement: Supplement 2. — Data Sharing Statement [file jamanetwopen-e253316-s002.pdf]

Data Sharing Statement

Schoenborn. Perspectives on AI and Novel Technologies Among Older Adults, Clinicians, Payers, Investors, and Developers. *JAMA Netw Open*. Published April 04, 2025. doi:10.1001/jamanetworkopen.2025.3316

Data
Data available: No

Additional Information
Explanation for why data not available: Because some of the participants refer to their specific professional roles throughout the interview, we cannot completely de-identify the participants without compromising the content of the data.
